# Supplementary material for: Genome-Wide Characterization of the Fur Regulatory Network Reveals a Link between Catechol Degradation and Bacillibactin Metabolism in Bacillus subtilis
Source: mBio. 2018 Oct 30;9(5):e01451-18. doi: 10.1128/mBio.01451-18 (PMC6212828; doi:10.1128/mBio.01451-18)
Supplement: FIG S1 [file mbo005184127sf1.docx]

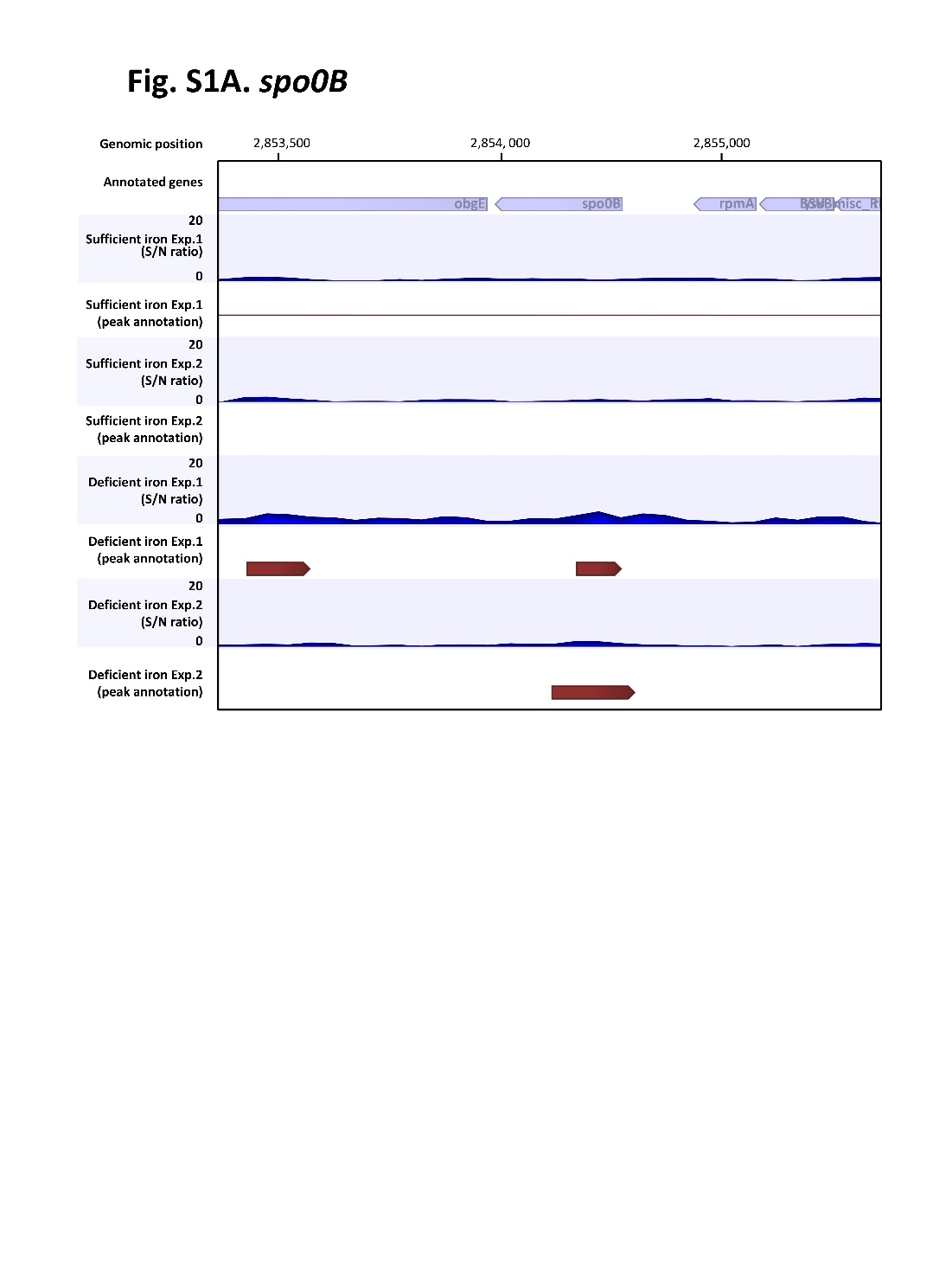


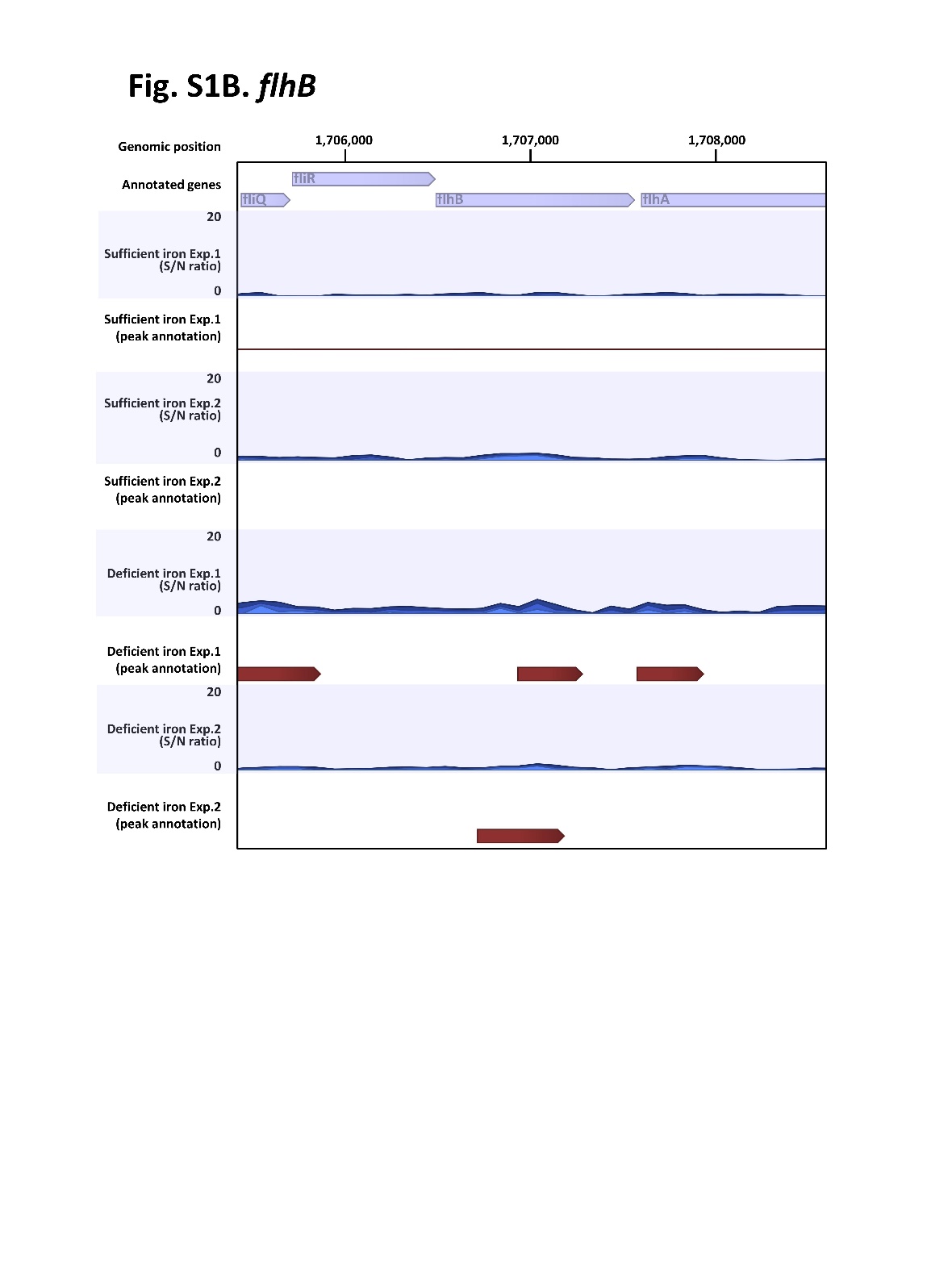


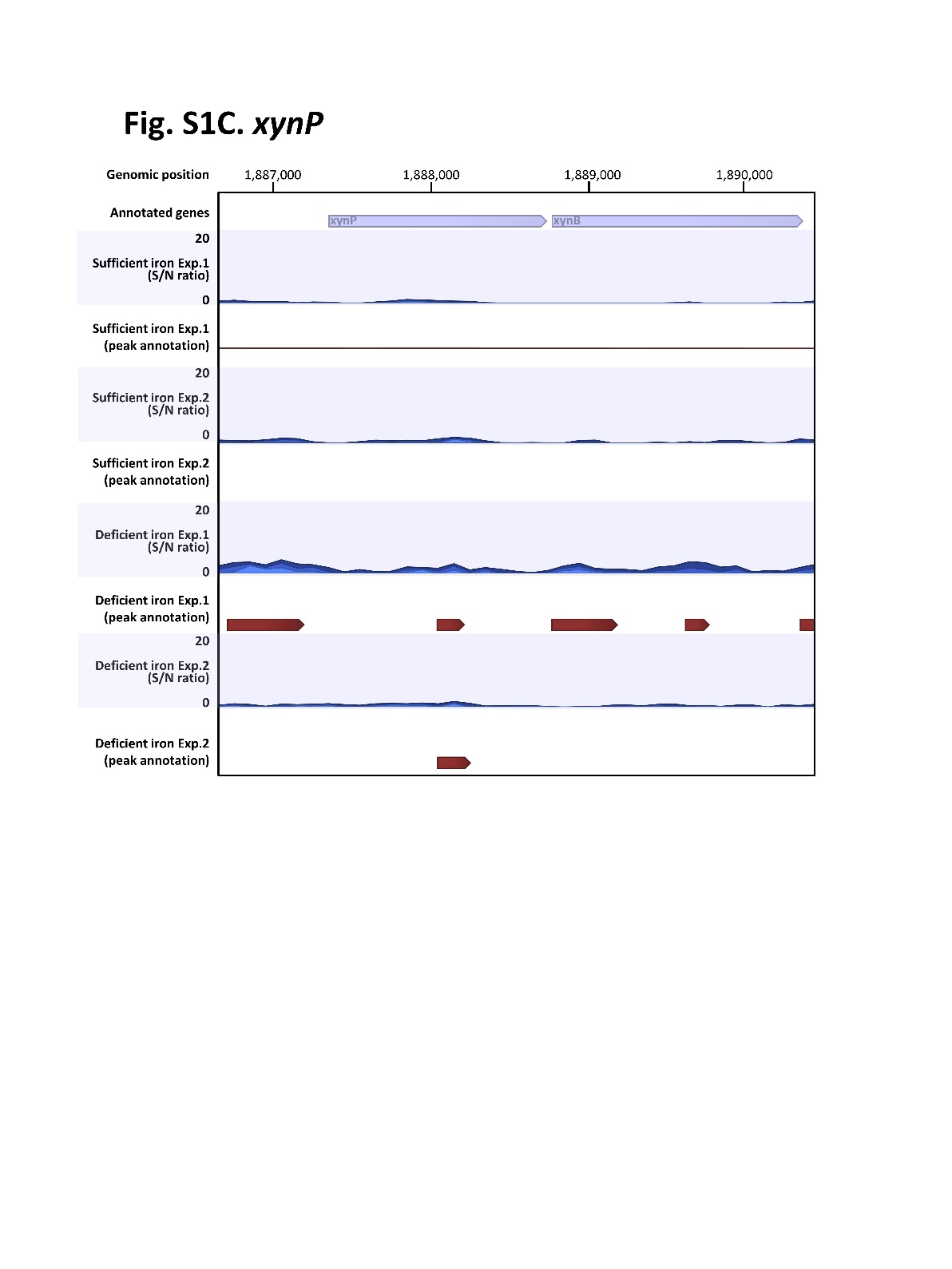


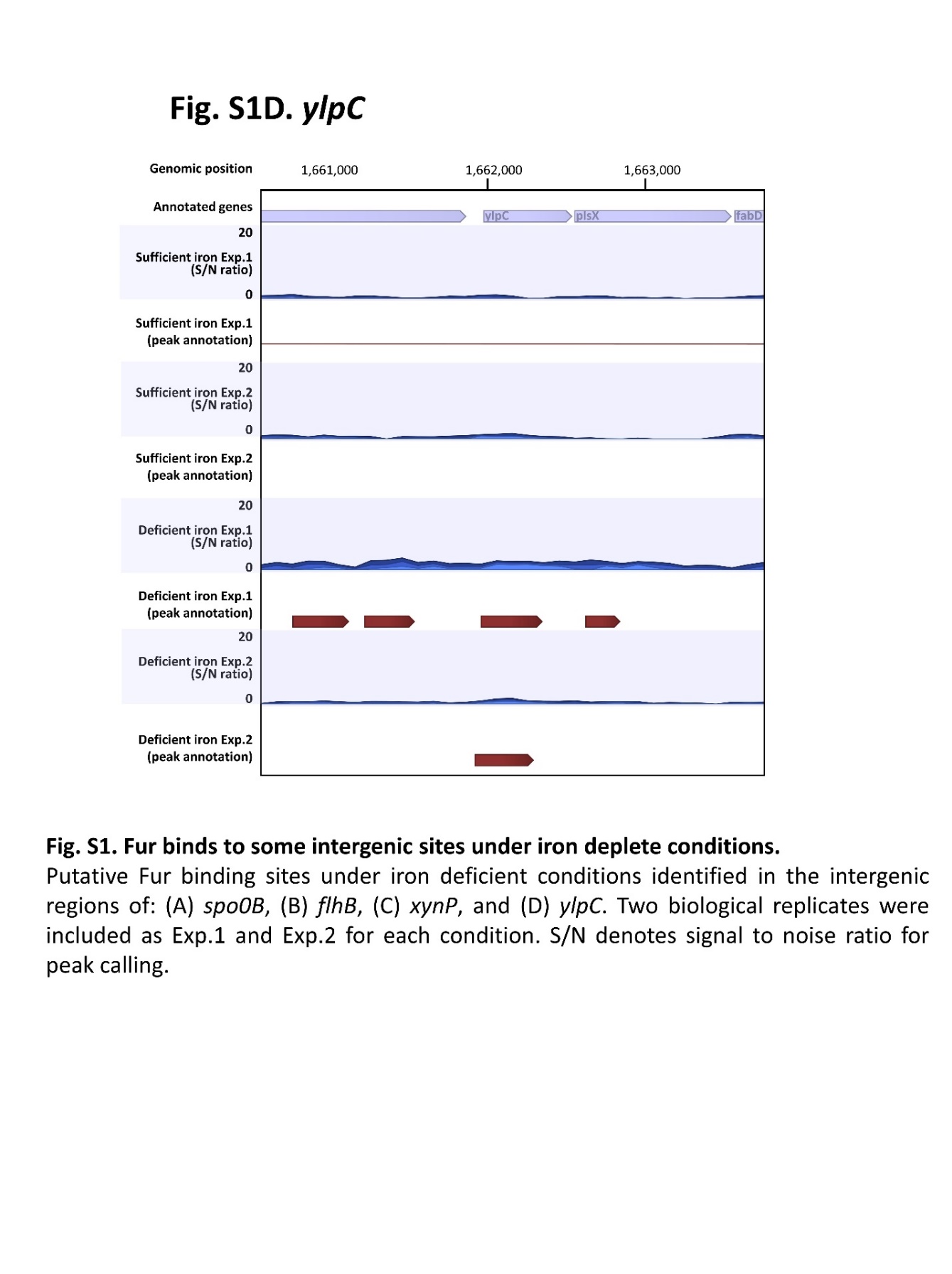


**Fig. S1. putative Fur binding sites specifically under iron deplete conditions.**

Putative apo-Fur binding sites identified in the intragenic regions of: (A) *spo0B*, (B) *flhB*, (C) *xynP*, and (D) *ylpC* (also known as *fapR*). Two biological replicates were included as Exp.1 and Exp.2 for each condition. S/N denotes signal to noise ratio for peak calling.
