# Supplementary material for: Genome-Wide Characterization of the Fur Regulatory Network Reveals a Link between Catechol Degradation and Bacillibactin Metabolism in Bacillus subtilis
Source: mBio. 2018 Oct 30;9(5):e01451-18. doi: 10.1128/mBio.01451-18 (PMC6212828; doi:10.1128/mBio.01451-18)
Supplement: FIG S2 [file mbo005184127sf2.docx]

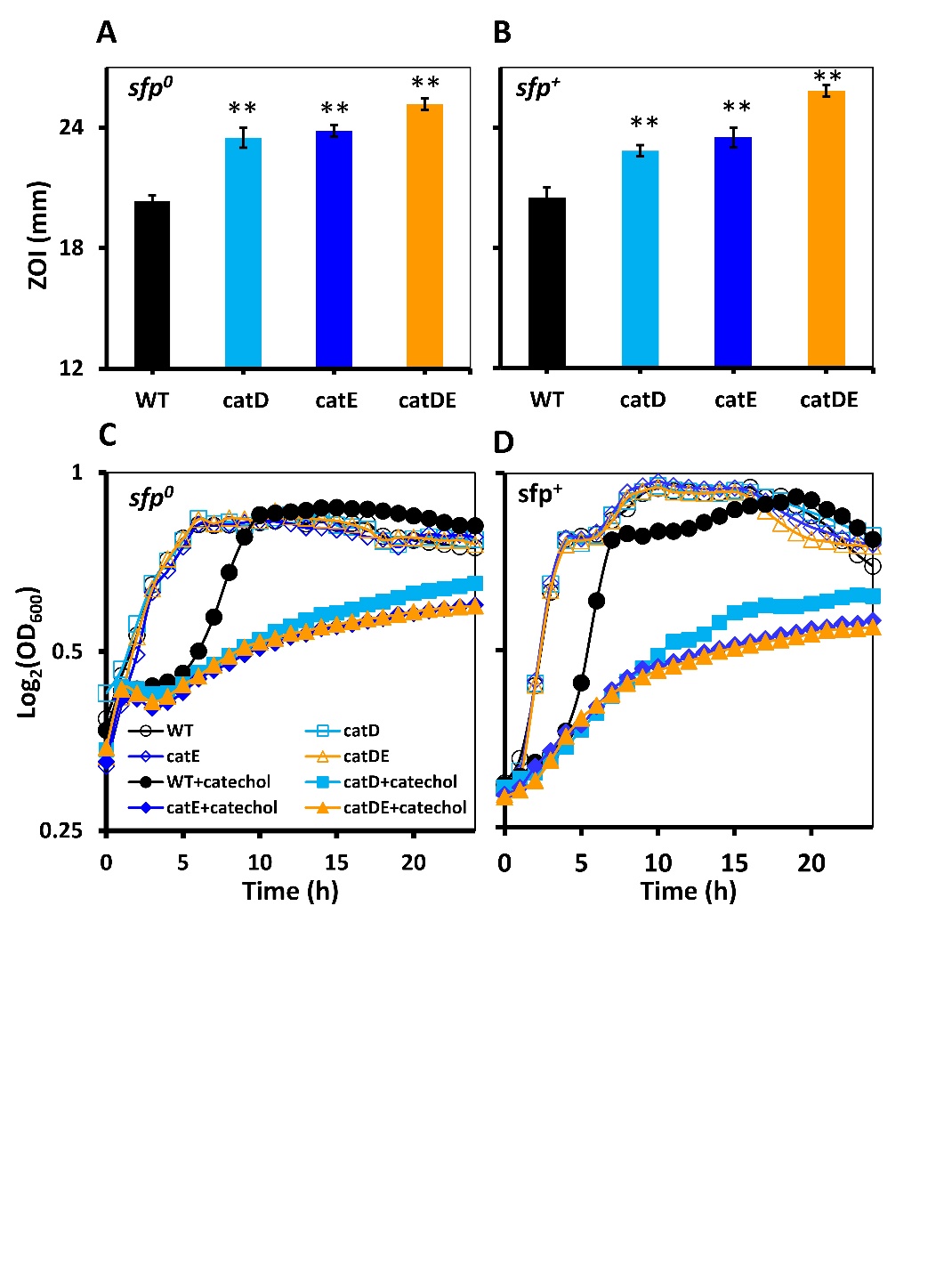


**Fig. S2. CatDE is critical for catechol degradation.**

Sensitivity of the *sfp^0^* (A) and *sfp^+^* strains (B) to catechol was evaluated in Belitsky minimal medium using a disk diffusion assay. 10 µl of 1 M catechol was applied to each disk. The data are expressed as the diameter (mean ± SEM; n = 3) of the inhibition zone (mm). Significant differences between wild type and mutant strains are indicated: ^**^P < 0.01.

Representative growth curves in Belitsky minimal medium with *sfp^0^* (C) and *sfp^+^* strains (D). 2mM catechol was used for both sets of experiments.
