# Supplementary material for: Genome-Wide Characterization of the Fur Regulatory Network Reveals a Link between Catechol Degradation and Bacillibactin Metabolism in Bacillus subtilis
Source: mBio. 2018 Oct 30;9(5):e01451-18. doi: 10.1128/mBio.01451-18 (PMC6212828; doi:10.1128/mBio.01451-18)
Supplement: FIG S3 [file mbo005184127sf3.docx]

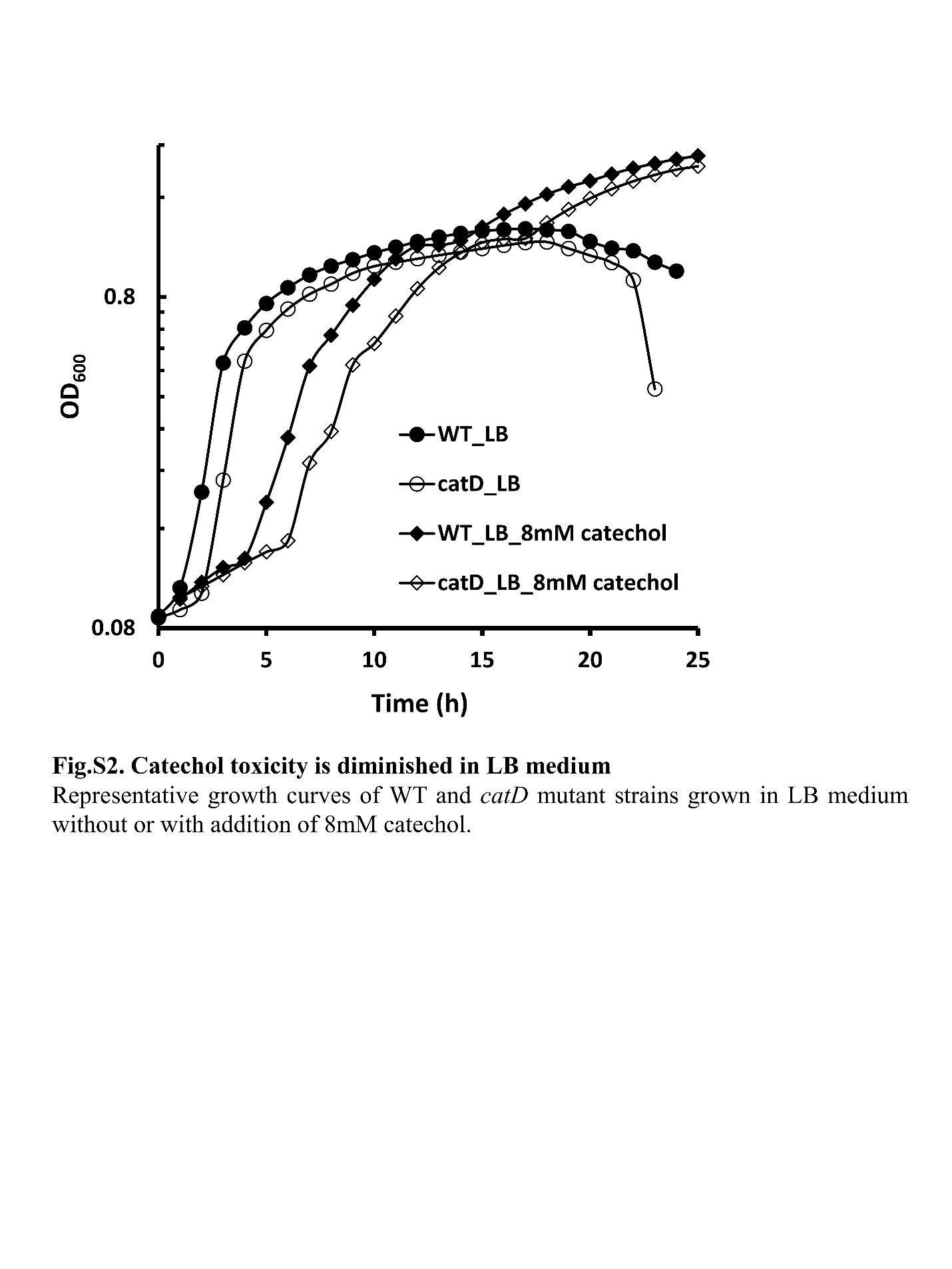


**Fig. S3. Catechol toxicity is diminished in LB medium**

Representative growth curves of WT and *catD* null mutant strains grown in LB medium without or with addition of 8mM catechol.
