# Supplementary material for: Genome-Wide Characterization of the Fur Regulatory Network Reveals a Link between Catechol Degradation and Bacillibactin Metabolism in Bacillus subtilis
Source: mBio. 2018 Oct 30;9(5):e01451-18. doi: 10.1128/mBio.01451-18 (PMC6212828; doi:10.1128/mBio.01451-18)
Supplement: FIG S4 [file mbo005184127sf4.docx]

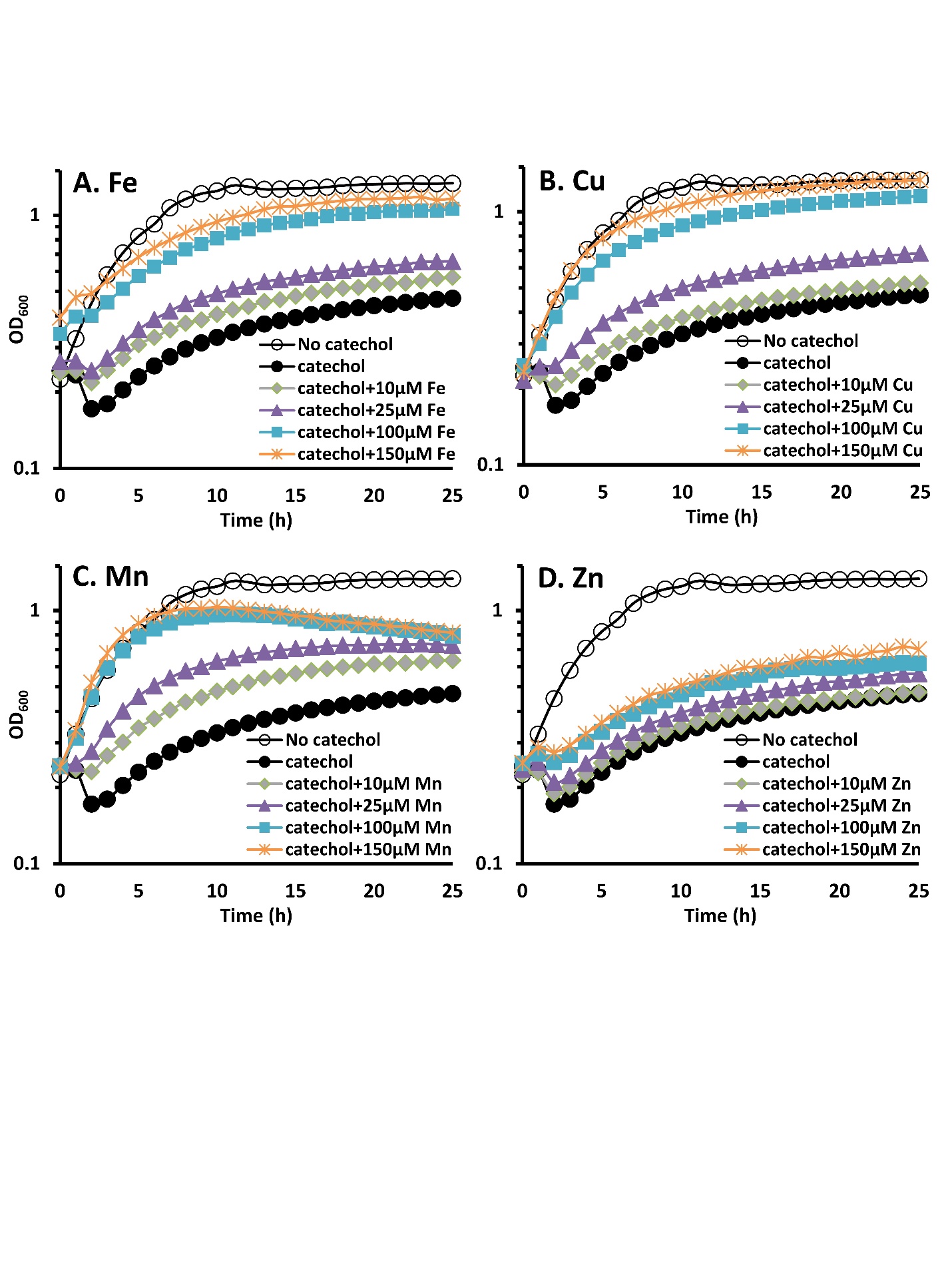


**Fig. S4. Metal-catechol complexes alleviates catechol intoxication**

Representative growth curves of *sfp^+^* *catD* mutant cells grown in Belitsky minimal medium without or with addition of 2mM catechol. To evaluate the effects of metal-catechol complexes on catechol intoxication, different concentrations of metal salts were tested: (A) FeSO_4_, (B) CuSO_4_, (C) MnCl_2_, and (D) ZnCl_2_. Metal-catechol complexes formed with iron, copper, and manganese alleviate catechol intoxication in a dose-responsive manner.
