# Supplementary material for: Genome-Wide Characterization of the Fur Regulatory Network Reveals a Link between Catechol Degradation and Bacillibactin Metabolism in Bacillus subtilis
Source: mBio. 2018 Oct 30;9(5):e01451-18. doi: 10.1128/mBio.01451-18 (PMC6212828; doi:10.1128/mBio.01451-18)
Supplement: TEXT S1 [file mbo005184127s1.docx]

**Text S1. Materials and Methods**

**Bacterial strains and growth conditions**

All strains used in the study are derivatives of *B. subtilis* strain CU1065 (WT), and were either *sfp^0^* or *sfp^+^* as indicated in Table S1. Cells were grown in LB or specified Belitsky minimal medium (1) with vigorous shaking or on solid agar plates (LB or Belitsky minimal medium) with appropriate antibiotic selection at 37^0^C. The concentrations of antibiotics used are: ampicillin (amp, 100 µg ml^-1^), spectinomycin (spec, 100 µg ml^-1^), tetracycline (tet, 5 µg ml^-1^), chloramphenicol (cm, 10 µg ml^-1^), kanamycin (kan, 15 µg ml^-1^), neomycin (neo, 8 µg ml^-1^), and macrolide lincosoamide-streptogramin B (MLS, 1 µg ml^-1^ erythromycin and 25 µg ml^-1^ lincomycin).

**Growth curves**

Cells were grown overnight in LB medium, subcultured at a 1:100 ratio into fresh LB medium or Belitsky minimal medium, and grown to early logarithmic phase (OD_600_~0.2-0.3). Cell Growth (OD_600_) was monitored every 15 min for 25 h using a Bioscreen growth analyzer (Growth Curves USA, Piscataway, NJ) at 37^0^C with continuous shaking. Data shown were representative growth curves and experiments were conducted at least three times with three biological replicates each time.

**RNA extraction and quantitative PCR (qPCR)**

Cells were grown at 37^0^C in LB medium overnight and subcultured at a 1:100 ratio into fresh LB medium. After OD_600_ reaches ~0.3-0.4, aliquots of 2 ml of cells were harvested 15 min after treatment or at different time points as indicated. Total RNA was extracted using RNeasy Mini Kit following the manufacturer’s instructions (Qiagen Sciences, Germantown, MD). All RNA samples were treated with Turbo-DNA free^TM^ DNase (Ambion^TM^) and precipitated with 2-3 volume of ethanol and 0.1 volume of 3M sodium acetate at -80^0^C overnight. RNA samples were washed with 70% ethanol and dissolved in nuclease-free water then quantified by NanoDrop spectrophotometer. Two hundred nanogram of total RNA from each sample was subjected to cDNA synthesis using high-capacity cDNA reverse transcription kits (Applied Biosystems, Foster City, CA). Primers used in this study are listed in Table S2. Quantitative PCR (qPCR) was then conducted using iQ SYBR green supermix in an Applied Biosystems 7300 Real Time PCR System. The housekeeping gene 23S rRNA was used as an internal control. Statistical analysis was carried out by the paired Student’s t-test using at least three independent replicates. *, *P* < 0.05 and **, P < 0.01.

**Disk diffusion assay**

Cells were grown overnight in LB medium and subcultured at 2% into Belitsky minimal medium to an OD_600_ of ~0.4. Cell culture (100 µl) was mixed with 4 ml of 0.75% Belitsky minimal medium agar and poured onto 1.5% Belitsky minimal medium agar plates. The plates were dried for 15 min at room temperature in a laminar flow hood. Filter paper disks (6.5 mm in diameter) soaked with 10 µl of 1 M catechol or 200 mM dipyridyl were placed on the top of the agar plates, and the plates were incubated at 37^0^C for 16–18 h. The data are expressed as the diameter (mean ± SEM; n=3) of the inhibition zone (mm). Statistically significant differences are determined by two-tailed *t-*test, *, *P* < 0.05 and **, P < 0.01.

**Chromatin immunoprecipitation coupled with high-throughput sequencing (ChIP-seq) and data analysis**

To obtain a genomic view of the Fur regulatory network *in vivo*, we performed chromatin immunoprecipitation coupled with high-throughput sequencing (ChIP-seq). A high-affinity ferrous iron efflux transporter FrvA from *Listeria monocytogenes* was utilized as an inducible genetic tool to impose iron limitation as described previously (2). *Bacillus* WT cells with C-terminal FLAG-tagged Fur at its native locus and an ectopic copy of *frvA* integrated at *amyE* locus were grown in LB medium amended with 25 µM iron to ensure Fur repression (2). After the cell culture reaches OD_600_ of ~0.25, an aliquot of cell culture was harvested to study Fur-dependent regulation under iron sufficient conditions. Then 1 mM IPTG was added to the cell culture to induce expression of FrvA to deplete intracellular iron pools. Fur loses its cofactor iron and falls off most of its targeted sites by 30 min as confirmed by ChIP-qPCR in the prior study (2). Thus cells were harvested at 30 min to study Fur-dependent regulation under iron deficient conditions. The ChIP-enriched DNA samples were prepared, purified, and quantified according to previous protocol (2). Experiments were performed in duplicates. Input DNA (no ChIP enrichment) was used as a negative control. Fifty to one hundred nanograms of ChIP or input DNA was used for DNA library preparation and then sequenced using an Illumina HiSeq 2500 at the Cornell University Life Sciences Core Laboratories Center. The sequence reads generated from ChIP-seq were trimmed and mapped onto the reference genome *B. subtilis* 168 (NC_000964) using CLC genomic workbench software version 8.5.1. A shape-based peak caller of the CLC genomic workbench (software version 8.5.1) was used to compare experiment alignments and control (input DNA) to identify ChIP peaks. The threshold for signal to noise ratio (S/N), which is analogous to ChIP-DNA enrichment ratio versus input DNA control, was set as 1.5 and the P-value threshold was set as 0.05. Peaks called in both independent samples (Exp. 1 and 2) were scored as real Fur ChIP peaks and listed in table S3-5. Some peaks with statistically significant S/N values in one replicate were also included in table S3-4. The sequence data was deposited to NCBI GEO repository (accession number: GSE119163).

**Electrophoretic mobility shift assay (EMSA)**

To validate whether Fur binding to the promoter region of *catDE* is sequence-specific, electrophoretic mobility shift assay (EMSA) was carried out using two different sets of DNA probes. The promoter region of *catD* along with part of its open reading frame (-155 to +386bp) were amplified by PCR and digested using HindIII. The purified digested products were used as one set of DNA probes and contain two fragments: a 323bp fragment (-155 to +168bp) encompassing the promoter region and a 218bp fragment located inside *catD* (+169 to +386bp), which serves as a negative control. The binding reaction was carried out as follows: ~ 180 ng of mixed DNA probes, 1 mM MnCl_2_, varied concentration of Fur protein (same preparation as analyzed previously for other target sites; 2), and 1X binding buffer (10 mM Tris-HCl, pH 8.0, 5% glycerol, 2 µg ml^-1^ salmon testes DNA, 50 mM NaCl, 1 mM DTT, 50 µg ml^-1^ BSA). The reaction was incubated at room temperature for 20 min and then subject to electrophoresis in a native 5% polyacrylamide gel using 40 mM TA buffer (pH 8.0, no EDTA). After electrophoresis, the gel was stained with ethidium bromide.

The second set of DNA probes includes one fragment containing the promoter region (-121 to +76bp) of *catD* and another within the open reading frame of *catD* (+137 to +278bp), which serves as a negative control. Both were amplified by PCR using specific primer sets listed in Table S2. Two hundred nanogram of purified DNA was labelled at 5’-ends with [γ-^32^P]-ATP using T4 polynucleotide kinase. After labelling, G10 column (NucAway^TM^ spin columns, Invitrogen) was used to remove the unincorporated (γ-^32^P) ATP and radioactivity of the probe was quantified by a scintillation counter. The binding reaction was carried out as follows: ~ 1 fmol of labelled DNA probe, 1 mM MnCl_2_, varied concentration of Fur protein, and 1X binding buffer (10 mM Tris-HCl, pH 8.0, 5% glycerol, 2 µg ml^-1^ salmon testes DNA, 50 mM NaCl, 1 mM DTT, 50 µg ml^-1^ BSA). The reaction was incubated at room temperature for 20 min and then subject to electrophoresis in a native 5% polyacrylamide gel using 40 mM TA buffer (pH 8.0, no EDTA). After electrophoresis, the gel was dried using a gel dryer, exposed to a phosphorimager screen overnight, and scanned by a phosphor image analyzer (Typhoon FLA 7000). The band intensity of unbound DNA was quantified using GelQuantNET software. The *K*_d_ value, corresponding to the concentration of Fur that gives rise to 50% half-maximal shifting of the DNA probe, was calculated using GraphPad Prism 5. The affinity of Fur binding to the promoter region of *dhbA* was also evaluated as a positive control (data not shown), which revealed a *K*_d_ value of <10 nM, consistent with prior results (2).

**Chromatin immunoprecipitation coupled with quantitative PCR (ChIP-qPCR)**

To monitor the occupancy of Fur at *catDE* operator site and other target sites *in vivo* (Fig. 9), we performed chromatin immunoprecipitation coupled with quantitative PCR according to the protocol as described previously (2). Briefly, cells (WT pMUTIN *:: fur-FLAG :: spec* and *amyE ::* P*_spac_*-*frvA*::*cm* pMUTIN *:: fur-FLAG :: spec*) were grown in LB medium amended with 25 µM FeSO­_4_ to OD_600_ of ~0.25, and 1 mM IPTG was added to cell culture to induce expression of FrvA as indicated. At different time points, 40 ml aliquots were harvested and the pellets were kept at −80 °C. The pellets were washed and resuspended with buffer CA (10 mM Na_2_HPO_4_, 2 mM KH_2_PO_4_, pH 7.4, 137 mM NaCl, and 2.7 mM KCl). The samples were incubated with 1% formaldehyde at room temperature for 10 min for crosslinking and then incubated with 133 mM glycine (pH7.5) at 4 °C for 30 min to quench the crosslinking.

To evaluate Fur occupancy at *catDE* operator site in WT and its derived mutant strains (Fig. 4), ChIP-qPCR was carried out. Cells were grown in LB medium at 37°C to an OD_600_ of ~0.4. The cell culture was neutralized with sodium phosphate (10 mM final, pH 7.4), and treated by formaldehyde (1% final) with agitation at 37°C for 5 min for crosslinking. Next, the cell culture was supplemented with ice-cold glycine (100 mM final, pH7.5), shifted to ice/water slurry, and then incubated with gentle rotating at 4 °C for 30 min to stop crosslinking.

After quenching the crosslinking, cells were spun down, washed twice with buffer CB (50 mM Tris–HCl pH 7.4, 150 mM NaCl and 1 mM EDTA), and then resuspended in 0.5 ml buffer CB followed by sonication for cell lysis and DNA fragmentation. The supernatant fraction was collected after centrifugation and the total protein concentration was quantified using a Bradford assay. Aliquots of 400 µg of total protein were kept at −80 °C. Aliquots of 1% volume of the lysate were diluted with CB buffer and kept at −80 °C to serve as the input-control (1% of input DNA). For immunoprecipitation, α-FLAG M2 magnetic agarose beads (Sigma, Cat# M8823) were washed and resuspended in 400 μl buffer CB. Aliquots of 400 µg of total protein were diluted, mixed with the washed magnetic beads, and incubated on a rotation mixer overnight in a cold room (4^0^C). The bead slurry was recovered by using a magnetic stand and washed twice with 500 μl of buffer CB. The protein–DNA complexes were eluted with 3X FLAG peptide according to the manufacture’s protocol. All samples including 1% input DNA samples were treated at 65^0^C for overnight to reverse crosslinking. Co-immunoprecipitated DNA was purified using a PCR purification Kit (Omega Biotek, Norcross, GA), quantified by NanoDrop spectrophotometer, and diluted appropriately followed by quantification using qPCR. Specific primer sets to the operator regions of the target genes are listed in Table S2. DNA enrichment was calculated based on the input DNA (1% of total DNA used for each ChIP experiment). The housekeeping gene *gyrA* was used as a non-specific negative control. Statistical analysis was carried out by the paired Student’s t-test using at least three independent replicates. *, *P* < 0.05 and **, P < 0.01.

**References**

1. **Miethke M, Schmidt S, Marahiel MA.** 2008. The major facilitator superfamily-type transporter YmfE and the multidrug-efflux activator Mta mediate bacillibactin secretion in *Bacillus subtilis*. J Bacteriol **190:**5143-5152.

2. **Pi H, Helmann JD.** 2017. Sequential induction of Fur-regulated genes in response to iron limitation in *Bacillus subtilis*. Proc Natl Acad Sci U S A doi:10.1073/pnas.1713008114.
