# Supplementary material for: Genome-Wide Characterization of the Fur Regulatory Network Reveals a Link between Catechol Degradation and Bacillibactin Metabolism in Bacillus subtilis
Source: mBio. 2018 Oct 30;9(5):e01451-18. doi: 10.1128/mBio.01451-18 (PMC6212828; doi:10.1128/mBio.01451-18)
Supplement: TABLE S1 [file mbo005184127st1.docx]

**Table S1. Strains and plasmids used in this study**

| **Strain** | **Genotype** | **Reference** |
| --- | --- | --- |
| **Strains constructed in *sfp^0^* background** | | |
| WT (CU1065) | *trpC2 attSPβ sfp^0^* | Lab stock |
| HB19396 | pMUTIN *:: fur-FLAG :: spec* | (1) |
| HB19398 | *amyE ::* P*_spac_*-*frvA*::*cm* pMUTIN *:: fur-FLAG :: spec* | (1) |
| HB17837 | *fur* :: *kan* | (2) |
| HB19414 | *narJ::mls* | This study |
| HB19415 | *yvlA::mls* | This study |
| HB19416 | *yufS::mls* | This study |
| HB19417 | yycE*::mls* | This study |
| HB19418 | *glxK::mls* | This study |
| HB19419 | *gntR::mls* | This study |
| HB19420 | *yybN::mls* | This study |
| HB19421 | *ppsB::mls* | This study |
| HB19422 | *ymcB::mls* | This study |
| HB19423 | *yhcJ::mls* | This study |
| HB19424 | *cspB::mls* | This study |
| HB19425 | *catD::mls* | This study |
| HB19426 | *ydeF::mls* | This study |
| HB19427 | *ybaC::mls* | This study |
| HB19428 | *ydeE::mls* | This study |
| HB19485 | *catE::mls* | This study |
| HB19488 | *catD markless* knockout (The *mls* resistance cassette looped out using pDR244, which is defined as KO) | This study |
| HB19491 | *catDE::mls* | This study |
| **Strains constructed in *sfp^+^* background** | | |
| HB5800 (WT) | *trpC2 attSPβ sfp^+^* | (3) |
| HB8247 | *fur* :: *kan* | (4) |
| HB19473 | *dhbA*::*spec* | This study |
| HB19474 | catD*::mls* | This study |
| HB19478 | *ymfD::mls* | This study |
| HB19481 | *ymfD* (KO) | This study |
| HB19490 | *catE::mls* | This study |
| HB19502 | *catDE::mls* | This study |
| HB19503 | *fur::kan catDE::mls* | This study |
| HB19505 | *fur::kan ymfD* (KO) | This study |
| HB19507 | *ymfD* (KO) *catDE::mls* | This study |
| HB19509 | *fur::kan ymfD* (KO) *catDE::mls* | This study |
| HB19511 | *ymfD* (KO) *dhbA*::*spec* | This study |
| HB19512 | *catDE::mls* *dhbA*::*spec* | This study |
| HB19513 | *fur::kan dhbA*::*spec* | This study |
| HB19514 | *ymfD* (KO) *catDE::mls* *dhbA*::*spec* | This study |
| HB19515 | *ymfD* (KO) *fur::kan dhbA*::*spec* | This study |
| HB19517 | *fur::kan catDE::mls* *dhbA*::*spec* | This study |
| HB19518 | *fur::kan catDE::mls* *dhbA*::*spec* *ymfD* (KO) | This study |
| HB19527 | *catR* (KO) | This study |
| HB19529 | *yodB* (KO) | This study |
| HB19531 | *fur::kan catR* (KO) | This study |
| HB19533 | *fur::kan yodB* (KO) | This study |
| HB19535 | *yodB::mls catR* (KO) | This study |
| HB19537 | *fur::kan yodB::mls catR* (KO) | This study |
| HB19539 | *catR::mls* | This study |
| HB19541 | *yodB::mls* | This study |
| HB19543 | *fur::kan catR::mls* | This study |
| HB19545 | *fur::kan yodB::mls* | This study |
| HB19547 | *besA::spec* | This study |
| HB19549 | *fur::kan besA::spec* | This study |
| HB19550 | *besA::spec ymfD* (KO) | This study |
| HB19552 | *fur::kan besA::spec ymfD* (KO) | This study |
| HB19554 | *fur::kan catDE::mls* *besA::spec* | This study |
| HB19556 | *catDE::mls* *besA::spec ymfD* (KO) | This study |
| HB19557 | *fur::kan catDE::mls* *besA::spec ymfD* (KO) | This study |
| HB19571 | *fur::kan ymfD* (KO) *catR::mls* | This study |
| HB19572 | *fur::kan ymfD* (KO) *yodB::mls* | This study |
| HB19573 | pMUTIN *:: fur-FLAG :: spec* | This study |
| HB19574 | *catR* (KO) pMUTIN *:: fur-FLAG :: spec* | This study |
| HB19575 | *yodB* (KO) pMUTIN *:: fur-FLAG :: spec* | This study |
| HB19576 | *catR* (KO) *yodB::mls* pMUTIN *:: fur-FLAG :: spec* |  |
| **Plasmid** | **Description** | **Reference** |
| pPL82 | Expression of gene under P*_spac_* promoter | Lab stock |
| pMUTIN | FLAG-tagged Fur in native locus | Lab stock |
| pDR244 | Generate markerless deletion using *cre*/lox-mediated loop-out system | Lab stock |

**References:**

1. **Pi H, Helmann JD.** 2017. Sequential induction of Fur-regulated genes in response to iron limitation in Bacillus subtilis. Proc Natl Acad Sci U S A doi:10.1073/pnas.1713008114.

2. **Baichoo N, Wang T, Ye R, Helmann JD.** 2002. Global analysis of the Bacillus subtilis Fur regulon and the iron starvation stimulon. Mol Microbiol **45:**1613-1629.

3. **Ollinger J, Song KB, Antelmann H, Hecker M, Helmann JD.** 2006. Role of the Fur regulon in iron transport in Bacillus subtilis. J Bacteriol **188:**3664-3673.

4. **Gaballa A, Helmann JD.** 2007. Substrate induction of siderophore transport in Bacillus subtilis mediated by a novel one-component regulator. Mol Microbiol **66:**164-173.
