# Supplementary material for: Genome-Wide Characterization of the Fur Regulatory Network Reveals a Link between Catechol Degradation and Bacillibactin Metabolism in Bacillus subtilis
Source: mBio. 2018 Oct 30;9(5):e01451-18. doi: 10.1128/mBio.01451-18 (PMC6212828; doi:10.1128/mBio.01451-18)
Supplement: TABLE S2 [file mbo005184127st2.docx]

**Table S2. Primer oligonucleotides**

| **Number** | **Name** | **Sequence** |
| --- | --- | --- |
| **Primers used for cloning** | | |
| 5782 | pPL82-check-for | AAGAAAGATATCCTAACAGCACA |
| 5783 | pPL82-check-rev | ACGATCTTTCAGCCGACTCA |
| 8095 | Fur-Flag-fwd | AGCGGATAACAATTAAGCTTCTCCTGAGATCGGTCTCGCTAC |
| 8096 | Fur-Flag-rev | CGATCGATAGCGCTGGTACCTTCAGTTTCTTTTCCGTTACAGC |
| 1452 | Spec-check-rev | CGTATGTATTCAAATATATCCTCCTCCTCAC |
| 1451 | mls-check-rev | gttttggtcgtagagcacacgg |
| 6554 | BKE_Scar_F | GCAGGCGAGAAAGGAGAGA |
| 6555 | BKE_Scar_R | CGAGGCTCCTGTCACTGCT |
| 8634 | CatD_Up_Fwd | GACAACCGTCACTAACCTTCTCG |
| 8635 | CatE_Down_Rev | AAGGTTTGGATTAATGACAGGATC |
| 8636 | YmfD_up_Fwd | GATACCATTTCTCCAGTGCTTGAA |
| 8637 | YmfD_Down_Rev | ATTATGACCAATTTTCAGCATCCG |
| 8638 | Fur_Up_Fwd | GGTCATTCTGTTTTTAGCGCTGA |
| 8639 | Fur_Up_Rev | TCGATCCTTTCCCTATGGTTCC |
| **Primers used for real-time qPCR** | | |
| 4368 | 23S-RT-F | AAAGGCACAAGGGAGCTTGACTGC |
| 4369 | 23S-RT-R | ATGAGCCGACATCGAGGTGCCAAA |
| 8600 | PpsB_RT_FW1 | TGATCAAGAACAGCAGACGCTGAT |
| 8601 | PpsB_RT_RV1 | TGCTGCCAAGCTGTACAGGTGAG |
| 8602 | PpsB_RT_FW2 | GAGAACCGTCACATCCAGCCTA |
| 8603 | PpsB_RT_RV2 | AGCAAGACCTAAAGCTGTCAGAAGG |
| 8604 | CspB_RT_FW | GGTTTCGGATTCATCGAAGTAGAAGG |
| 8605 | CspB_RT_RV | TAACGTTAGCAGCTTGTGGTCCG |
| 8606 | YhcJ_RT_FW | CGGAGTCTGATATCGCACTTGCTA |
| 8607 | YhcJ_RT_RV | CGCGATATATCTCGGATTCGTTC |
| 8608 | GidA_RT_FW | CCATCCATTGAAGACAAAGTTGTCC |
| 8609 | GidA_RT_RV | TTGCATCATACTCGATCGCATAGC |
| 8610 | TufA_RT_FW | ACCAGGTACAATCACTCCACACAG |
| 8611 | TufA_RT_RV | TCTCCAGGCATAACCATTTCTACG |
| 8612 | YbaC_RT_FW | GTTCATTGGGATCAGAGAGGATCG |
| 8613 | YbaC_RT_RV | ACCTGGCTGATTCCATAATACGTG |
| 8614 | CatD_RT_FW | CCATCCAATTTTTCGGCAGCAT |
| 8615 | CatD_RT_RV | CCGATCAGTGTAAGAGCGAACAG |
| 8616 | YmcB_RT_FW | GGATTGGGAGACGGAAGAAAGTTCT |
| 8617 | YmcB_RT_RV | CCGAGCTCACCAAACACCTTGT |
| 8618 | YdeE_RT_FW | CGTCGCAGACGTCTTACTCTTGC |
| 8619 | YdeE_RT_RV | TGTTAGCTGGAAGGTCATTCGTGG |
| 8620 | YdeF_RT_FW | AGGGCTGCTGAGTGCGACTGTA |
| 8621 | YdeF_RT_RV | CAAGGGCTCATATGAAGCGTGATCT |
| 8622 | NarJ_RT_FW | TGGAAGGCTCTTATCCAAGAAATCG |
| 8623 | NarJ_RT_RV | TCCCTTTGCTCGCCTGAGTTAAAG |
| 8624 | YvlB_RT_FW | AACCGTCTTTAGGAGCCAAGCTG |
| 8625 | YvlB_RT_RV | GCAGCTCAACACTGGAAAAGTCAG |
| 8626 | YybN_RT_FW | TTCCTTCTTGCGGCTGCTTTAG |
| 8627 | YybN_RT_RV | GTACCTTTACCTTCTGCCGATTTCTG |
| 8628 | YxaA_RT_FW | TCTCTGGTCGATGCGACCAATG |
| 8629 | YxaA_RT_RV | CTTATCTACAGGCACCAGATGCAGC |
| **Primers used for synthesis of EMSA probes** | | |
| 8040 | dhbA_fwd | GTCACTGAAATTATATTTGACTG |
| 8041 | dhbA_rev | ATCATCAATTCCTTTCTTCGCTC |
| 8632 | CatD_EMSA_Fw | TAGACCTTTGTCCTGCACAGAG |
| 8633 | CatD_EMSA_Rev | CTCTTAAAAGTAATGTGCCAATTTC |
| **Primers used for ChIP-qPCR** | | |
| 8083 | dhbA_CHIP_Fwd | TGACGGACCGCATCTATCAATGG |
| 8084 | dhbA_CHIP_rv | AGCTTCGCCTATTCCTTGGGC |
| 8093 | YwbL_CHIP_fwd | GACAAAGGACAGGAACTGGCTATG |
| 8094 | YwbL_CHIP_rv | CGAGCCATCATGTTCCTCCTATAA |
| 8092 | FsrA_CHIP_fwd | CGATTGACATTGATACTGAGAATCA |
| 8089 | FsrA_CHIP_rv | GAACAGAGAGTAGCTTCTCTCTAT |
| 8632 | CatD_EMSA_Fw | TAGACCTTTGTCCTGCACAGAG |
| 8633 | CatD_EMSA_Rev | CTCTTAAAAGTAATGTGCCAATTTC |
