# Supplementary material for: Genome-Wide Characterization of the Fur Regulatory Network Reveals a Link between Catechol Degradation and Bacillibactin Metabolism in Bacillus subtilis
Source: mBio. 2018 Oct 30;9(5):e01451-18. doi: 10.1128/mBio.01451-18 (PMC6212828; doi:10.1128/mBio.01451-18)
Supplement: TABLE S3 [file mbo005184127st3.docx]

**Table S3. Known Fur targets associated with ChIP-peaks.**

| **Gene or**  **operon** | **Function** | **S/N ratio^*^** | | | | **P-value^#^** | | | | **fur/WT**  **mRNA ratio^‡^** |
| --- | --- | --- | --- | --- | --- | --- | --- | --- | --- | --- |
|  |  | **Iron sufficient** | | **Iron deficient** | | **Iron sufficient** | | **Iron deficient** | |  |
|  |  | **Exp.1** | **Exp.2** | **Exp.1** | **Exp.2** | **Exp.1** | **Exp.2** | **Exp.1** | **Exp.2** |  |
| *feuABC-ybbA* | bacillibactin uptake | 53.6 | 47.2 | 55.1 | 24.6 | 0 | 0 | 0 | 1.8E-134 | 24.3 |
| *fhuBGC* | hydroxamate siderophore uptake | 48.4 | 38.5 | 20.5 | 11.1 | 0 | 0 | 4.4E-94 | 1E-28 | 9.8 |
| *fhuD* | hydroxamate siderophore uptake | 48.4 | 38.5 | 20.5 | 11.1 | 0 | 0 | 4.4E-94 | 1E-28 | 9.8 |
| *dhbACEBF* | bacillibactin biosynthesis | 45.9 | 37.4 | 20.7 | 10.6 | 0 | 2.3E-306 | 1.9E-95 | 2.2E-26 | 166.7 |
| *btr* | transcription activator of bacillibactin uptake system | 44.3 | 38.2 | 18.0 | 9.5 | 0 | 0 | 1E-72 | 8.8E-22 | 8.8 |
| *yoaJ* | bacterial expansin, required for the colonization of maize roots | 42.0 | 32.6 | 27.8 | 14.9 | 0 | 2.0E-233 | 7.6E-171 | 2.7E-50 | 4.0 |
| *ykuNOP* | flavodoxin, replaces ferredoxin under iron limited conditions | 37.6 | 29.3 | 5.7 | 3.9 | 0 | 3.3E-189 | 5.6E-09 | 4.2E-05 | 74.2 |
| *yfhC* | unknown | 36.4 | 29.0 | 9.1 | 4.5 | 7.9E-290 | 1.1E-184 | 5.3E-20 | 3.1E-06 | 2.0 |
| *fbpAB* | RNA chaperone for fsrA | 32.3 | 21.7 | 2.8 | ND | 3.6E-229 | 9.9E-105 | 0.0027 | ND | ND^§^ |
| *fecCDEF* | Fe-citrate ABC transporter | 31.3 | 18.8 | 5.0 | 2.8 | 1.0E-214 | 6.9E-79 | 3.3E-07 | 0.0028 | 2.7 |
| *fbpC* | RNA chaperone for fsrA | 29.7 | 26.6 | 7.1 | 4.1 | 1.4E-196 | 3.5E-156 | 8.7E-13 | 2.0E-05 | ND^§^ |
| *hmoA* | heme monooxygenase | 23.5 | 19.6 | 4.1 | 1.5 | 1.1E-122 | 4.7E-86 | 2.3E-05 | 0.06 | 5.9 |
| *efeUOB* | elemental iron uptake | 21.8 | 19.5 | 3.2 | ND | 5.4E-106 | 8.6E-85 | 6.7E-04 | ND | 3.2 |
| *yxeB* | hydroxamate siderophore | 20.9 | 17.0 | ND | ND | 6.6E-97 | 8.2E-65 | ND | ND | 17.1 |
| *yhfQ* | Fe-citrate uptake | 19.0 | 12.6 | 2.9 | 2.5 | 9.1E-81 | 1.7E-36 | 0.002 | 0.006 | 4.5 |
| *yusV* | ABC transporter for the siderophores | 14.7 | 19.3 | ND | ND | 2.7E-49 | 6.7E-83 | ND | ND | 2.2 |
| *besA* | trilactone hydrolase, catalyses ferri-bacillibactin hydrolysis | 14.6 | 9.1 | 2.7 | ND | 1.2E-48 | 6.6E-20 | 0.003 | ND | 24.7 |
| *fsrA* | iron sparing response | 11.7 | 10.2 | 2.6 | 2.1 | 4.6E-32 | 1.2E-24 | 0.005 | 0.02 | ND^§^ |
| *yusU* | unknown | 6.7 | 2.3 | 3.4 | ND | 7.8E-12 | 0.01 | 0.0003 | ND | 2.1 |
| *ywiE-ywjAB* | minor cardiolipin synthetase, protect against paraquat stress | 4.7 | 2.4 | ND | ND | 1.2E-6 | 7.8E-3 | ND | ND | 1.1 |
| *ycgT* | NADPH:ferredoxin oxidoreductase | 4.1 | 3.2 | ND | ND | 2.6E-5 | 1.8E-4 | ND | ND | 0.8 |
| *ypbNOPQ* | petrobactin ABC transporter | 3.1 | 3.5 | ND | ND | 9.9E-4 | 2.7E-4 | ND | ND | 2.6 |
| *yfiY* | ABC transporter (binding protein) for the siderophore schizokinen and arthrobactin | 1.9 | 2.6 | ND | ND | 0.03 | 4.3E-3 | ND | ND | 10.6 |
| *yfiZ-yfhA* | siderophore (schizokinen and arthrobactin) transport | 1.9 | 2.6 | ND | ND | 0.03 | 4.3E-3 | ND | ND | 2.1 |
| *nasBCDEF* | nitrate reductase | 1.6 | 1.6 | ND | ND | 0.05 | 0.05 | ND | ND | 0.8 |
| *S477-ykoP* | unknown | 0 | 0 | 3.2 | 1.3 | ND | ND | 8.1E-4 | 0.09 | ND^§^ |

Note: ^*^S/N ratio denotes signal to noise ratio for peak calling. Two biological replicates are included for each condition as Exp.1 and Exp.2. ^#^The P-value threshold was set as 0.05. ^‡^*fur*/WT mRNA ratio refers to the mRNA abundance of the target gene or the first gene in each operon in a *fur* mutant compared to wild type strain, which is taken from prior microarray data (1). ND, not detectable. ND^§^, not determined since no oligos were designed for these sites in the microarray analysis (1).

**Reference**

1. **Baichoo N, Wang T, Ye R, Helmann JD.** 2002. Global analysis of the Bacillus subtilis Fur regulon and the iron starvation stimulon. Mol Microbiol **45:**1613-1629.
