# Supplementary material for: Genome-Wide Characterization of the Fur Regulatory Network Reveals a Link between Catechol Degradation and Bacillibactin Metabolism in Bacillus subtilis
Source: mBio. 2018 Oct 30;9(5):e01451-18. doi: 10.1128/mBio.01451-18 (PMC6212828; doi:10.1128/mBio.01451-18)
Supplement: TABLE S4 [file mbo005184127st4.docx]

**Table S4. putative Fur-regulated genes associated with ChIP-peaks**

| **Gene** | **Function** | **S/N ratio*** | | | | **P-value^#^** | | | | | **Δ*fur*/WT**  **Expression ratio^‡^** | **Distance (bp) relative to start codon^§^** |
| --- | --- | --- | --- | --- | --- | --- | --- | --- | --- | --- | --- | --- |
|  |  | **Iron sufficient** | | **Iron deficient** | | **Iron sufficient** | | | **Iron deficient** | |  |  |
|  |  | **Exp.1** | **Exp.2** | **Exp.1** | **Exp.2** | **Exp.1** | **Exp.2** | | **Exp.1** | **Exp.2** |  |  |
| **ChIP-peaks located at intragenic regions** | | | | | | | | | | | | |
| *ppsB* | Plipastatin synthetase | 21.5 | 22.0 | 5.0 | 2.8 | 9.8E-103 | 4.2E-107 | | 2.5E-7 | 0.003 | 0.3 | +3297 |
| *gidA* | tRNA modification | 13.3 | 4.8 | 3.3 | 2.9 | 8.3E-41 | 8.6E-7 | | 5.0E-4 | 0.002 | 0.7 | +276 |
| *tufA* | elongation factor Tu | 12.6 | 3.9 | 4.4 | 1.6 | 1.7E-36 | 4.4E-5 | | 4.6E-6 | 0.06 | 0.7 | +655 |
| *yycE* | unknown | 9.4 | 4.2 | 3.1 | 1.8 | 3.3E-21 | 1.2E-5 | | 8.9E-4 | 0.03 | 0.7 | +181 |
| *ywmF* | unknown | 8.0 | 5.3 | 4.6 | 1.5 | 6.1E-16 | 4.7E-8 | | 2.1E-6 | 0.07 | 1.2 | +254 |
| *ydcC* | unknown | 7.8 | 2.2 | 4.5 | ND | 4.1E-15 | 0.015 | | 3.5E-6 | ND | 0.7 | +702 |
| *copA* | copper export | 6.6 | 2.9 | ND | ND | 1.9E-11 | 0.0018 | | ND | ND | 0.05 | +1232 |
| *ydfB* | unknown | 6.1 | 2.6 | ND | ND | 5.2E-10 | 0.004 | | ND | ND | 0.9 | +329 |
| *tagF* | biosynthesis of teichoic acid | 5.8 | 2.0 | ND | ND | 2.9E-9 | 0.02 | | ND | ND | 1.2 | +728 |
| *kinB* | initiation of sporulation | 5.2 | 4.7 | 2.5 | ND | 9.1E-8 | 1.1E-6 | | 0.006 | ND | 1.7 | +206 |
| *wapA* | cell wall-associated protein precursor | 5.0 | 2.6 | 2.6 | ND | 2.6E-7 | 4.8E-3 | | 0.004 | ND | 3.2 | +3210 |
| *gltT* | glutamate/aspartate uptake | 4.8 | 4.0 | ND | ND | 5.4E-7 | 3.4E-5 | | ND | ND | 1.2 | +190 |
| *ywiE* | phospholipiid biosynthesis | 4.7 | 2.4 | ND | ND | 1.2E-6 | 0.008 | | ND | ND | 1.2 | +483 |
| *ydgE* | unknown | 4.4 | 1.9 | 3.6 | ND | 4.5E-6 | 0.03 | | 1.7E-4 | ND | 1.5 | +141 |
| *bdbD* | thiol-disulfide oxidoreductase | 4.1 | 1.9 | ND | ND | 2.0E-5 | 0.03 | | ND | ND | 0.8 | +511 |
| *pucL* | urate oxidase, purine utilization | 3.9 | 2.0 | ND | ND | 4.5E-5 | 0.02 | | ND | ND | 1.0 | +791 |
| *yxjB* | unknown | 3.7 | 1.7 | ND | ND | 9.7E-5 | 0.05 | | ND | ND | 0.5 | +226 |
| *ydbT* | confers resistance to antimicrobials | 3.7 | 2.0 | ND | ND | 1.1E-4 | 0.02 | | ND | ND | 0.7 | +107 |
| *cimH* | citrate uptake | 3.6 | 2.1 | 3.0 | ND | 1.4E-4 | 0.02 | | 0.001 | ND | 0.4 | +614 |
| *ytxC* | unknown | 3.6 | 1.8 | ND | ND | 1.4E-4 | 0.04 | | ND | ND | 1.2 | +699 |
| *tlpB* | chemotaxis control | 3.6 | 1.8 | ND | ND | 1.9E-4 | 0.04 | | ND | ND | 3.1 | +1155 |
| *yvdP* | outer spore coat protein | 3.2 | 2.3 | ND | ND | 7.7E-4 | 0.01 | | ND | ND | 1.0 | +988 |
| *rapG* | response regulator aspartate phosphatase | 3.1 | 4.1 | ND | ND | 9.5E-4 | 1.7E-5 | | ND | ND | 0.6 | +181 |
| *hutI* | imidazolone-5-pro-pionate hydrolase | 3.0 | 2.2 | ND | ND | 0.002 | 0.01 | | ND | ND | 1.1 | +773 |
| *yesX* | rhamnogalacturonan lyase | 2.9 | 1.8 | ND | ND | 0.002 | 0.03 | | ND | ND | 1.1 | +578 |
| *ywbI* | transcriptional regulator | 2.8 | 3.9 | ND | ND | 0.003 | 5.2E-5 | | ND | ND | 0.7 | +559 |
| *yydJ* | export of the mature YydF epipeptide | 2.7 | 2.3 | 2.5 | 1.5 | 0.003 | 0.01 | | 0.005 | 0.06 | 1.6 | +547 |
| *cycB* | uptake of galactotriose | 2.7 | 1.9 | 2.3 | ND | 0.003 | 0.03 | | 0.01 | ND | 1.1 | +102 |
| *bioF* | biosynthesis of biotin | 2.7 | 2.0 | ND | ND | 0.003 | 0.02 | | ND | ND | 0.9 | +574 |
| **ChIP peaks located at regulatory regions** | | | | | | | | | | | | |
| *yhcJ* | unknown | 13.4 | 11.1 | ND | ND | 2.8E-41 | 1.0E-28 | | ND | ND | 1.3 | -110 |
| *cspB* | major cold-shock protein | 13.4 | 11.1 | ND | ND | 2.8E-41 | 1.0E-28 | | ND | ND | 0.3 | -331 |
| *catD* | catechol detoxification | 10.1 | 14.0 | ND | ND | 3.5E-24 | 1.2E-44 | | ND | ND | 3.3 | -49 |
| *ymcB* | tRNA methyl-thiotransferase | 8.9 | 8.3 | 2.9 | 1.3 | 1.8E-19 | 7.3E-17 | | 0.002 | 0.09 | 0.7 | -90 |
| *ydeE* | transcription regulator | 8.8 | 12.7 | ND | ND | 6.0E-19 | 2.4E-37 | | ND | ND | 0.6 | +41 |
| *ydeF* | transcription regulator | 8.8 | 12.7 | ND | ND | 6.0E-19 | 2.4E-37 | | ND | ND | 0.6 | -273 |
| *yvlB* | unknown | 7.8 | 2.9 | 3.8 | ND | 2.9E-15 | 0.002 | | 6.2E-5 | ND | 0.4 | -96 |
| *narJ* | chaperone for the nitrate reductase | 6.8 | 3.4 | 2.5 | ND | 5.5E-12 | 0.0003 | | 0.006 | ND | 0.2 | -282 |
| *yddT* | unknown | 6.4 | 1.6 | ND | ND | 6.4E-11 | 0.05 | | ND | ND | 1.3 | -15 |
| *S1211-mrpA* | Na /H antiporter | 5.9 | 1.9 | 3.1 | ND | 1.9E-9 | 0.03 | | 0.001 | ND | 1.4 | -79 |
| *yufS* | unknown | 5.9 | 1.9 | 3.1 | ND | 1.9E-9 | 0.03 | | 0.001 | ND | 1.1 | -163 |
| *gntR* | regulation of gluconate utilization | 5.5 | 3.4 | 5.0 | 3.5 | 1.5E-8 | 0.0003 | | 2.9E-7 | 2.8E-4 | 0.9 | -202 |
| *glxK* | putative glycerate kinase | 5.5 | 3.4 | 5.0 | 3.5 | 1.5E-8 | 0.0003 | | 2.9E-7 | 2.8E-4 | 1.7 | -2 |
| *S1564-yybN* | unknown | 5.3 | 2.7 | ND | ND | 6.1E-8 | 0.003 | | ND | ND | 1.7 | -290 |
| *ydcC* | unknown | 4.6 | 2.2 | ND | ND | 2.4E-6 | 0.015 | | ND | ND | 0.7 | -25 |
| *glmS* | glutamine-fructose-6-phosphate transaminase | 4.5 | 1.7 | ND | ND | 3.6E-6 | 0.04 | | ND | ND | 0.7 | -236 |
| *S1529 -yxcE* | unknown | 4.2 | 2.3 | ND | ND | 1.3E-5 | 0.01 | | ND | ND | 1.0 | -335 |
| *ydbL* | unknown | 4.1 | 2.4 | ND | ND | 2.5E-5 | 0.008 | | ND | ND | 0.6 | -50 |
| *yceF* | general stress protein | 4.1 | 2.0 | 2.4 | ND | 2.5E-5 | 0.02 | | 0.007 | ND | 0.5 | -223 |
| *S124*  *-nucA* | catalyzes DNA cleavage during transformation | 3.8 | 4.7 | ND | ND | 8.6E-5 | 1.3E-6 | | ND | ND | 3.5 | -188 |
| *S1501*  *-yxjB* | unknown | 3.7 | 1.9 | 3.6 | ND | 1.2E-4 | 0.03 | | 1.5E-4 | ND | 0.5 | -145 |
| *yvaC* | unknown | 3.5 | 1.7 | 2.7 | ND | 1.9E-4 | 0.04 | | 0.003 | ND | 1.5 | -68 |
| *S1458* | new RNA feature | 3.5 | 2.3 | 3.5 | ND | 2.5E-4 | 0.01 | | 2.2E-4 | ND | ND^†^ | -133 |
| *sufC* | synthesis of Fe-S clusters | 3.5 | 1.7 | ND | ND | 2.5E-4 | 0.04 | | ND | ND | 1.0 | -39 |
| *ybyB* | general stress protein | 3.2 | 2.1 | ND | 1.5 | 7.3E-4 | 0.02 | | ND | 0.07 | 0.7 | -218 |
| *ybeC* | similar to amino acid transporter | 3.2 | 2.1 | ND | 1.5 | 7.3E-4 | 0.02 | | ND | 0.07 | 1.3 | -390 |
| *ahpF* | alkyl hydroperoxide reductase | 3.1 | 1.8 | 3.2 | ND | 8.8E-4 | 0.04 | | 7.1E-4 | ND | 1.7 | -54 |
| *cdd* | pyrimidine interconversion | 3.1 | 2.4 | 3.6 | 1.5 | 9.5E-4 | 0.009 | | 1.5E-4 | 0.07 | 1.5 | -352 |
| *S1550*  *-yycN* | putative N-acetyltransferase | 3.1 | 4.1 | ND | ND | 9.5E-4 | 1.7E-5 | | ND | ND | 1.7 | -312 |
| *ycbU* | cysteine desulfurase | 3.0 | 1.7 | 2.3 | ND | 0.001 | 0.04 | | 0.01 | ND | 1.5 | -53 |
| *pcp* | Pyrrolidone carboxylate peptidase | 3.0 | 1.7 | 2.3 | ND | 0.001 | 0.04 | | 0.01 | ND | 1.1 | -261 |
| *ywoB* | unknown | 2.9 | 5.3 | ND | ND | 0.002 | 5.7E-8 | | ND | ND | 1.2 | -127 |
| *amtB* | ammonium transporter (or *nrgA*) | 2.9 | 5.3 | ND | ND | 0.002 | 5.7E-8 | | ND | ND | 0.6 | -155 |
| *S1216 -degQ* | regulation of exoenzyme synthesis | 2.9 | 3.3 | ND | ND | 0.002 | 5.4E-4 | | ND | ND | 2.9 | -259 |
| *yuzC* | spore coat protein | 2.9 | 3.3 | ND | ND | 0.002 | 5.4E-4 | | ND | ND | 1.2 | -209 |
| *S1435 -rpmE* | ribosomal protein L31 | 2.9 | 2.4 | ND | ND | 0.002 | 0.008 | | ND | ND | 0.3 | -68 |
| *mrgA* | Iron storage protein | 2.9 | 1.8 | 2.9 | ND | 0.002 | 0.04 | | 0.002 | ND | 0.1 | -203 |
| *katA* | main vegetative catalase 1 | 2.8 | 1.8 | 2.7 | 1.6 | 0.002 | 0.04 | | 0.004 | 0.06 | 0.3 | -87 |
| *S309* | new RNA feature | 2.8 | 1.8 | 2.7 | 1.6 | 0.002 | 0.04 | | 0.004 | 0.06 | ND^†^ | +64 |
| *yczK* | unknown | 2.8 | 2.6 | ND | ND | 0.003 | 0.004 | | ND | ND | ND^†^ | -327 |
| *yceG* | general stress protein | 2.7 | 2.6 | 2.6 | ND | 0.003 | 0.005 | | 0.005 | ND | 0.5 | -75 |
| *iolE* | myo-inositol catabolism | 2.6 | 1.7 | ND | ND | 0.004 | 0.04 | | ND | ND | 0.6 | -331 |
| *yhdB* | unknown | 2.6 | 1.7 | ND | ND | 0.005 | 0.05 | | ND | ND | 1.3 | -55 |
| *yhdC* | unknown | 2.6 | 1.7 | ND | ND | 0.005 | 0.05 | | ND | ND | 0.9 | -256 |
| *fer* | ferredoxin | 2.6 | 2.8 | 2.8 | ND | 0.005 | 0.003 | | 0.003 | ND | 0.5 | -183 |
| *ypbB* | unknown | 2.6 | 2.8 | 2.8 | ND | 0.005 | 0.003 | | 0.003 | ND | 1.4 | -88 |
| *ynzG* | unknown | 2.5 | 1.8 | 3.3 | ND | 0.006 | 0.04 | | 4.5E-4 | ND | 0.3 | -8 |
| *yqxI* | unknown secreted protein | ND | 5.2 | 2.8 | ND | ND | 1.1E-7 | | 0.003 | ND | 1.0 | -162 |
| *S974-S975* | new RNA feature | ND | 5.2 | 2.8 | ND | ND | 1.1E-7 | | 0.003 | ND | ND^†^ | +36 |
| **putative binding sites associated with specific apo-Fur ChIP-peaks** | | | | | | | | | | | | |
| *Spo0B* | sporulation initiation phosphotransferase | ND | ND | 3.7 | 1.6 | ND | ND | 1.0E-4 | | 0.05 | 0.62 | +124 |
| *flhB* | part of flagellar Type III secretion system | ND | ND | 3.5 | 1.6 | ND | ND | 2.1E-4 | | 0.05 | 1.0 | +553 |
| *xynP* | beta-xyloside permease | ND | ND | 2.8 | 1.5 | ND | ND | 0.002 | | 0.05 | 0.19 | +761 |
| *ylpC* | Transcription repressor of fatty acid biosynthesis, also known as *fapR* | ND | ND | 2.6 | 1.6 | ND | ND | 0.004 | | 0.05 | 3.6 | +154 |

Note: ^*^S/N ratio denotes signal to noise ratio for peak calling. Two biological replicates are shown for each condition as Exp.1 and Exp.2. ^#^The P-value threshold was set as 0.05. ^‡^*fur*/WT expression ratio refers to the mRNA abundance of the target gene in a *fur* mutant compared to wild type strain, which is taken from prior microarray data (1). ND, not detectable. ND^†^, not determined since no oligos were designed for these sites in the microarray analysis (1). ^§^Distance (bp) relative to start codon refers to the distance between the start codon of target gene and the center of each ChIP peak.

**Reference**

1. **Baichoo N, Wang T, Ye R, Helmann JD.** 2002. Global analysis of the Bacillus subtilis Fur regulon and the iron starvation stimulon. Mol Microbiol **45:**1613-1629.
