# Supplementary material for: Genome-Wide Characterization of the Fur Regulatory Network Reveals a Link between Catechol Degradation and Bacillibactin Metabolism in Bacillus subtilis
Source: mBio. 2018 Oct 30;9(5):e01451-18. doi: 10.1128/mBio.01451-18 (PMC6212828; doi:10.1128/mBio.01451-18)
Supplement: TABLE S5 [file mbo005184127st5.docx]

| **Genes** | **Function** | **Putative Fur box*** | **mRNA ratio (*fur*/WT) (microarray)^#^** | **mRNA ratio (*fur*/WT)**  **(qPCR)^§^** | **mRNA ratio**  **(WT_high_iron_ vs WT_control_)^§^** | **mRNA ratio**  **(WT_dipyridyl_ vs WT_control_)^§^** | **Sensitivity to**  **high iron** | **Sensitivity to dipyridyl** |
| --- | --- | --- | --- | --- | --- | --- | --- | --- |
| *ppsB* | plipastatin synthetase | TGAGAAGCGTTCTCA | 0.3 | 1.7 | 0.7 | 1.0 | NS | NS |
| *gidA* | tRNA modification | half-boxes | 0.7 | 1.6 | 0.9 | 0.9 | NS | + |
| *tufA* | Elongation factor Tu | Half-boxes | 0.7 | 1.4 | 0.9 | 1.2 | NS | NS |
| *ybaC* | putative proline  iminopeptidase | Half-boxes | 1.0 | 0.6 | 0.8 | 0.7 | NS | NS |
| *yycE* | unknown | Half-boxes | 0.7 | ND | ND | ND | NS | NS |
| *yhcJ* | similar to ABC transporter  (binding lipoprotein) | TGACAACGTTTATCA | 1.3 | 0.9 | 0.8 | 0.6 | NS | + |
| *cspB* | major cold-shock protein  (RNA chaperone) | TGACAACGTTTATCA | 0.3 | 1.0 | 0.9 | 0.8 | NS | + |
| *catD* | catechol detoxification | TGATAACATTTATCA | 3.3 | 5.3 | 1.0 | 2.6 | NS | + |
| *ymcB* | tRNA  methylthiotransferase | TGATTATTATTCTCA | 0.7 | 1.2 | 1.3 | 1.0 | NS | NS |
| *ydeE* | putative transcription  factor (AraC family) | TGATATTGATTTTCA | 0.6 | 1.5 | 1.0 | 1.0 | NS | NS |
| *ydeF* | transcriptional regulator  (MocR/ GabR family) | TGATATTGATTTTCA | 0.6 | 1.6 | 0.9 | 1.0 | NS | NS |
| *yvlB* | unknown | TGATAATGCCAATGA | 0.4 | 1.2 | 0.5 | 0.7 | NS | NS |
| *narJ* | chaperone for the nitrate  reductase | TGACAGACATTTTCT | 0.2 | 0.3 | 2.8 | 0.3 | NS | + |
| *yufS* | unknown | TGATTTTAAATATAC | 1.1 | ND | ND | ND | NS | NS |
| *gntR* | Regulation of gluconate  utilization | TGAAAATCATCATTG | 0.9 | ND | ND | ND | NS | NS |
| *glxK* | putative glycerate kinase | TGAAAATCATCATTG | 1.7 | ND | ND | ND | NS | NS |
| *yybN* | unknown | TGAAAATCAAATCA  ATAAAAATATTCTCA | ND^†^ | 1.5 | 1.0 | 0.8 | NS | + |

**Table S5. putative Fur target genes evaluated in this study**

Note: ^*^Putative Fur box was identified within some ChIP peaks and the nucleotide bases highlighted in red are mismatches compared with the minimal 7-1-7 consensus sequence (1); ^#^mRNA ratio (*fur*/WT) refers to the mRNA abundance of the target gene in *fur* null mutant compared to wild type strain, which was taken from prior microarray data (2); ^§^mRNA ratio refers to the mRNA levels of the target gene in different genetic background or different conditions as indicated, which was obtained by qPCR; ND, not determined; ND^†^, not determined since no oligos were designed for this site in the microarray analysis (2). NS, not significant; +, more sensitive to the compound tested in null mutant compared to WT.

**Reference**

1. **Baichoo N, Helmann JD.** 2002. Recognition of DNA by Fur: a reinterpretation of the Fur box consensus sequence. J Bacteriol **184:**5826-5832.

2. **Baichoo N, Wang T, Ye R, Helmann JD.** 2002. Global analysis of the Bacillus subtilis Fur regulon and the iron starvation stimulon. Mol Microbiol **45:**1613-1629.
